# Acute Post-Streptococcal Glomerulonephritis in the Northern Territory of Australia: A Review of Data from 2009 to 2016 and Comparison with the Literature

**DOI:** 10.4269/ajtmh.18-0093

**Published:** 2018-11-05

**Authors:** Swasti Chaturvedi, Rowena Boyd, Vicki Krause

**Affiliations:** 1 Department of Paediatrics, Royal Darwin Hospital, Darwin, Australia;; 2 Department of Health, Centre for Disease Control, Darwin, Australia

## Abstract

Acute post-streptococcal glomerulonephritis (APSGN) is an inflammatory kidney disease following infection with nephritogenic strains of Group A *Streptococcus*. In 1991, APSGN became notifiable in the Northern Territory (NT) of Australia with cases recorded on the NT Notifiable Disease Database (NTNDS). The case definition of a confirmed case requires laboratory definitive evidence or laboratory suggestive evidence in conjunction with a clinically compatible illness. Probable cases require clinical evidence only. Acute post-streptococcal glomerulonephritis notifications from 2009 to 2016 were extracted from the NTNDS. Of the 322 cases, 261 were confirmed and 61 probable. The majority, 304 (94%), were Aboriginal and the median age was 8 years (range: 0–62 years). Incidence for confirmed cases was 13.8/100,000 person-years, with inclusion of probable cases increasing incidence to 17.0/100,000 person-years. Highest incidence of confirmed cases was in Aboriginal children less than 15 years of age at 124.0 cases/100,000 person-years. The rate ratio of confirmed cases in Aboriginal to non-Aboriginal Australians was 18.9 (95% confidence interval: 11.4–33.6). Recent trends show a consistently high number of notifications annually with less frequent outbreaks. The Aboriginal population of the NT continues to have high rates of APSGN with recent trends showing higher rates than previously reported. Sustained preventative efforts and continued surveillance strategies are needed.

## Introduction

Aboriginal Australians suffer one of the highest rates of chronic kidney disease (CKD) in the world.^1^ Acute post-streptococcal glomerulonephritis (APSGN) is an inflammatory kidney disease caused by prior infection with nephritogenic strains of Group A *Streptococcus* (GAS). Previous work by Hoy et al.^2^ reported APSGN as a major risk factor for the development of CKD in the Australian Aboriginal population living in remote communities.

Recognition of the heavy burden of disease in the Northern Territory (NT) led to establishment of case definitions, mandatory clinician notification of disease, and establishment of an APSGN database in 1991. The purpose of notification includes facilitating accurate disease monitoring and directed public health response. The confirmed case requires either laboratory definitive evidence (renal biopsy suggestive of APSGN) or the presence of both laboratory suggestive evidence and clinical evidence (Table 1). A contact is defined as those staying in the house in the 2 weeks preceding the onset of the illness.

**Table 1:** Case definition for notification of APSGN in the Northern Territory

Confirmed case	Probable case	
A confirmed case requires either:	A probable case requires clinical evidence only.	
1. Laboratory definitive evidence or		
2. Laboratory suggestive evidence and clinical evidence.		
Laboratory definitive evidence	Laboratory suggestive evidence	Clinical evidence At least two of the following Facial edema ≥ Moderate hematuria on dipstick Hypertension Peripheral edema
Renal biopsy suggestive of APSGN.	1. Hematuria on microscopy (RBC > 10/μL) and	
	2. Evidence of recent streptococcal infection (positive Group A streptococcal culture from skin or throat, or elevated ASO titer or Anti-DNase B) and	
	3. Reduced C3 level.	

APSGN = acute post-streptococcal glomerulonephritis.

The epidemiology of APSGN in the NT from 1991 to 2008 has previously been reported.^3^ This article describes the epidemiology of APSGN in the NT from 2009 to 2016 and identifies trends. A review of the literature enables a comparison of NT disease burden against global trends.

## Methods

### Setting.

In 2016, the estimated resident population in the NT was 228,833.^4^ Aboriginal Australians constitute approximately 30% of the population, and 80% of the NT Aboriginal population live in remote or very remote locations. The Top End of the NT has two seasons: the cooler dry season which runs from April to September and the hot, rainy, and humid wet season from October to March. In Central Australia, there is a desert climate with intermittent rain throughout the year and cooler weather from April to September (mean temperature range 4–28°C) than the months of October to March (mean temperature range 15–36°C).^5^

### Epidemiological data.

Data were collected as previously described, with the period of study identified from notifications between 1991 and 2016.^3^ This study concentrates on describing post-2008 data, a previously undescribed period. Table 1 describes the case definition of a confirmed and probable case. Reoccurrence of disease was identified through matching at least two of the following notification fields including first name, surname, and date of birth. An outbreak year was declared if the number of notifications in the NT for a particular year more than doubled the mean number of notifications for the preceding 4 years.

### Ethics approval.

Ethics approval was obtained from the Human Research Ethics Committee, Menzies School of Health Research. HREC 2017-2914.

### Statistical analysis.

Population data for incidence calculations were obtained from the Australian Bureau of Statistics and NT Department of Health Analysis and reported as a rate per 100,000 person-years. Unless stated otherwise, calculations for incidence were based on confirmed cases only.

### Review criteria.

We searched Medline, Ebsco, and Pubmed using the terms (glomerulonephritis AND (strep OR streptococcal OR post-streptococcal OR poststreptococcal OR Group A Strep)) AND (studies OR study). The search was restricted to articles published in English language after 1965. We also searched reference lists of nephrology textbooks, review articles, and all articles and relevant reviews identified.

## Results

### Northern Territory data.

Between 2009 and 2016, there were 261 confirmed and 61 probable cases of APSGN for a total of 322 cases. Characteristics of notifications are shown in Table 2, with most of the cases in Aboriginal Australians (304/322, 94%) and those less than 15 years of age (277/322; 86%). Median age was 8 years, with an interquartile range of 1–44 years including one infant less than 12 months diagnosed at 11 months.

**Table 2:** Characteristics of cases of acute post-streptococcal glomerulonephritis in the Northern Territory, 2009–2016

Characteristic	*n*/Total 322	%
Aboriginal	304	94%
Non-Aboriginal	18	6%
Age (years)	8 (median)	0–62 (range)
0–4	93	29%
5–9	115	36%
10–14	69	21%
15–19	10	3%
Older than 19	10	3%
Male	178	55%
Hospitalized	280/304*	92%

* Denominator only available for 304 cases.

**Figure 1.**
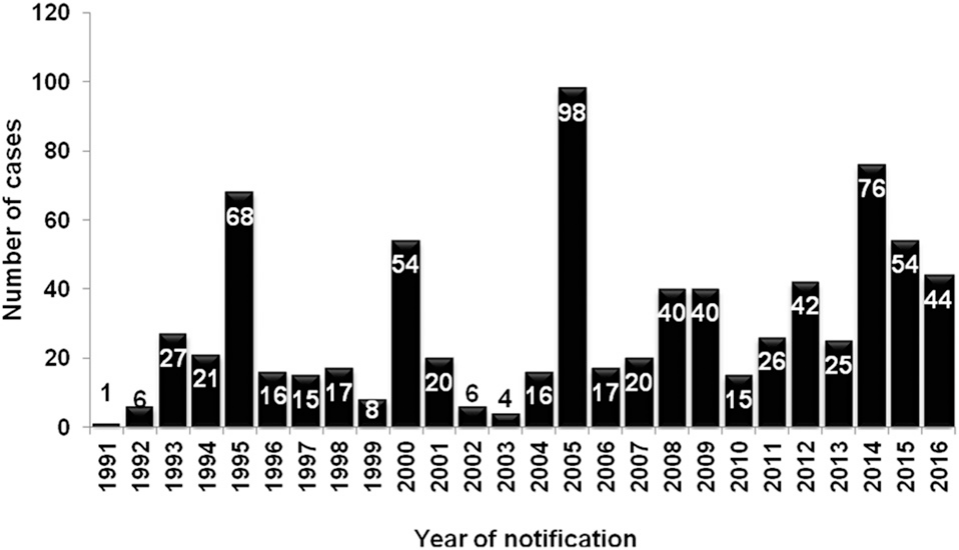
Epidemic curve of notified acute post-streptococcal glomerulonephritis cases, Northern Territory, 1991–2016.^32^

For non-outbreak years, an upward trend in annual notifications has been observed since 2008 (Figure 1). Previous trends of five yearly outbreaks before 2006 have not been evident in recent years. An outbreak of 76 notifications occurred in 2014.

**Figure 2.**
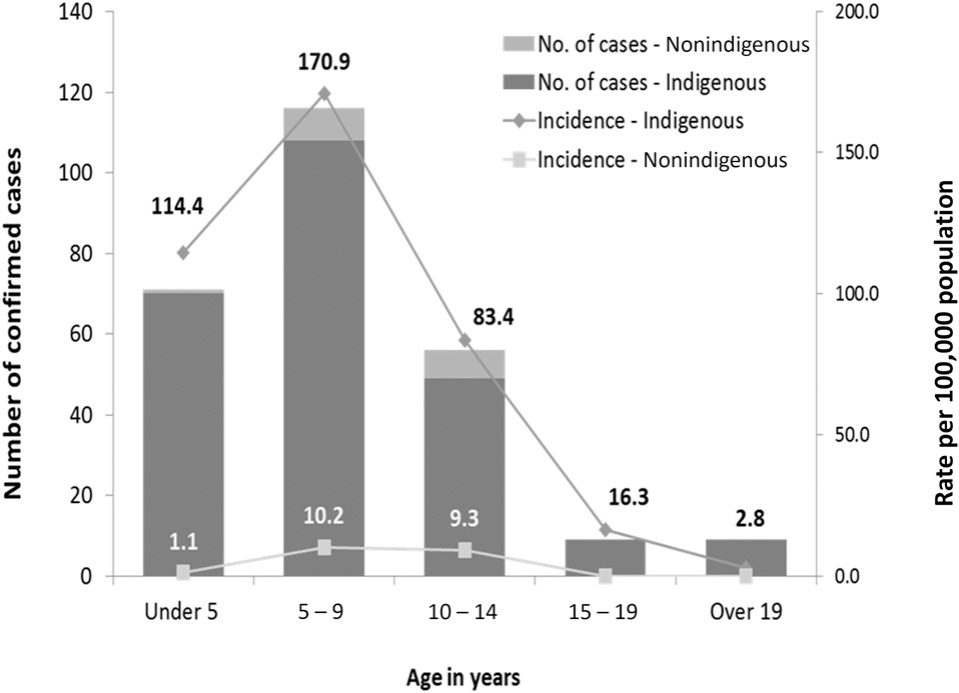
Notifications (*n* = 322) of confirmed acute post-streptococcal glomerulonephritis and annualized incidence per 100,000 person-years by age group, Northern Territory, 2009–2016.

The frequency and incidence of APSGN notifications by age group are shown in Figure 2. Highest incidence of APSGN was observed in Aboriginal children less than 15 years of age, with 124.0 (95% confidence interval [CI]: 108.4–141.2) cases per 100,000 (Table 3). For non-Aboriginal children less than 15 years of age, annualized incidence was 7.4 (95% CI: 3.7–10.6) cases per 100,000, resulting in an incidence rate ratio of 18.9 (95% CI: 11.4–33.6).

**Table 3:** Incidence of confirmed acute post-streptococcal glomerulonephritis in Northern Territory, Australia (Annual incidence per 100,000 person-years)

	Age (years)	1992–2007 (95% CI)^3^	2009–2016 (95% CI)
Total population	All ages	12.5 (11.3–13.8)	13.8 (12.1–15.5)
	0–14	41.4 (37.0–46.1)	56.9 (50.0–64.5)
	> 14	2.1 (1.6–2.8)	1.2 (0.7–1.9)
Aboriginal	All ages	39.7 (35.7–44.0)	43.6 (38.3–49.4)
	0–14	94.3 (84.2–105.3)	124.0 (108.4–141.2)
	> 14	7.3 (5.3–9.9)	4.8 (2.8–7.5)
Non-Aboriginal	All ages	0.74 (0.42–1.2)	1.2 (0.7–1.9)
	0–14	2.3 (1.2–4.2)	6.6 (3.7–10.6)
	> 14	0.30 (0.095–0.69)	0 (0.0–0.3)

CI = confidence interval.

**Figure 3.**
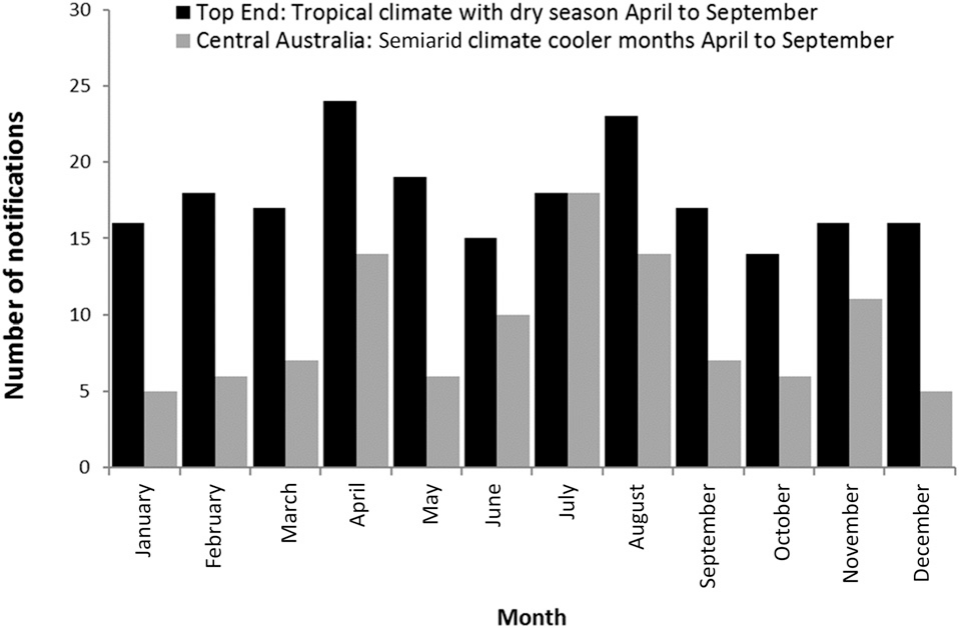
Acute post-streptococcal glomerulonephritis notifications by month, Top End and Central Australia, Northern Territory, 2009–2016.

Notifications in the Top End (tropical climate) were similar, regardless of seasons with 116/213 (54%) notifications occurring in the dry season, April–September (95% CI: 48–61%) (Figure 3). In Central Australia (semiarid climate), notifications were significantly higher during the months April–September, with 69/109 (63%) cases occurring during these winter months (95% CI: 54–72%).

Between 1991 and 2016, there were 10 people reported with a second episode of APSGN. Time between diagnoses ranged from 210 days to 7 years (mean 4 years). During the same period, two sets of twins were notified with APSGN, with a difference of 6 and 10 days between diagnosis dates for the respective twin.

### Literature review.

We found 15 studies reporting data on incidence of APSGN as shown in Table 4.^3,6–20^ Highest incidence was reported among Aboriginal Australians aged < 15 years (239 per 100,000)^9^ and the lowest in an Italian study (0.09 per 100,000).^10^

**Table 4:** Incidence of APSGN (reference numbers: ^3, 6–19^)

Study (reference number)	Age group (years)	Period of study	Country	APSGN incidence (per 100,000 per year)
Present study	All ages	2009–2016	NT, Australia	Overall: 17.0
				Aboriginal < 15 years of age: 155.1
				Non-Aboriginal < 15 years of age: 7.4
Wong et al.^18^	< 14	2007–2009	New Zealand	Overall: 9.7
				Pacific Islander: 45.5
				Maori: 15.7
				European/other: 2.6
Marshall et al.^3^	All ages	1991–2008	NT, Australia	Overall: 12.5
				Aboriginal < 15 years of age: 94.3
				Non-Aboriginal < 15 years of age: 2.3
Becquet et al.^7^	< 15	2005–2007	French Polynesia	18
Berrios et al.^8^	< 15	1980–1999	Chile	1980–1983: 6.2
				1984–1989 (epidemic outbreak): 13.2
				1990–1999: 1.7
Herrera and Rodri'guez-Iturbe^12^	All ages	1991–1998	Venezuala	Goajiro Indians: 2.9
Muscatello et al.^16^	< 20	1989–1998	New South Wales, Australia	2.2
Baker et al.^6^	5–14	1988–1998	New Zealand	Maori: 48
				Pacific Islander: 80
Carapetis^9^	< 15	1993–1995	NT, Australia	Aboriginal: 239;
				Non-Aboriginal: 6
Coppo et al.^10^	All ages	1998	Italy	< 60 years: 0.04
				> 60 years: 0.09
Eke and Eke^11^	< 15	1986–1991	Nigeria	24.3
Simon et al.^17^	All ages	1986–1990	France	0.15
Yap et al.^19^	< 12	1985	Singapore	10.8
Lennon et al.^14^	Children	1981–1984	New Zealand	Maori: 50.5
				Pacific Islander: 46.5
				Other: 5.9
Majeed et al.^15^	Children	1980–1983	Kuwait	17.8
Khuffash et al.^13^	Children	1980–1984	Kuwait	19.5

APSGN = acute post-streptococcal glomerulonephritis; NT = Northern Territory.

## Discussion

The prognosis of APSGN is relatively benign in the low-risk populations; however, it is associated with a 5- to 6-fold increased risk of CKD in resource-limited settings.^2,21^ The incidence of APSGN has declined globally in the past four decades and particularly in some developed countries.^10,17^ Acute post-streptococcal glomerulonephritis, however, remains an important health problem in developing countries and tropical regions of a number of developed countries. Some developing countries report APSGN as the most common cause of acute nephritis.^22,23^ Carapetis et al. analyzed data from 11 population-based studies and calculated the annual incidence of APSGN as ranging from 6 to 24.3 per 100,000 person-years in the more developed to the less developed countries, respectively.^24^ The true incidence of the disease is difficult to determine because of the under-recognition of the milder cases of APSGN and the transient nature of the illness. Indeed, the subclinical cases are thought to be 4–19 times more common than symptomatic disease.^25,26^ Thus, we assume that incidence is higher than reported.

Marshall et al.^3^ have previously reported 16 years data on APSGN from the NT of Australia. They reported 415 confirmed cases and 23 probable cases during the study period of 1991 to July 2008. The current data reveal that the NT of Australia continues to have a heavy burden of APSGN with 261 confirmed and 61 probable cases between 2009 and 2016.

Incidence in children less than 15 years of age has significantly increased in both Aboriginal and non-Aboriginal children. Before 2009, Marshall et al. identified an incidence in Aboriginal children less than 15 years of age at 94.3 per 100,000 person-years (95% CI: 84.2–105.3) compared with our more recent finding of 124.0 person-years (95% CI: 108.4–141.2). In non-Aboriginal children, incidence has increased from 2.3 (95% CI: 1.2–4.2) for the 1991–2008 period to 6.6 (95% CI: 3.7–10.6) cases per 100,000. It is not known if there is a real increase in the number of cases or greater reporting of cases. Interestingly, the Aboriginal to non-Aboriginal rate ratio in the same age group has significantly decreased from 53.6 (95% CI: 32.6–94.8) to 18.9 (95% CI: 11.4–33.6) pre- and post-2009. We do not know if this decreasing gap between disease burden in Aboriginal and non-Aboriginal children represents greater burden of disease in non-Aboriginal children or greater identification and reporting of APSGN in this population.

The previous trends of five yearly outbreaks are not evident since 2005, with the outbreak in 2014 “overdue,” occurring 9 years after the previous outbreak of 98 cases in 2005. Rather, recent trends show a consistently higher number of notifications annually with less frequency of outbreaks. Not enough time has elapsed to determine if this is a long-term trend.

The seasonal variation noted in the present study contrasts with that reported by Marshall et al.^3^ Previously, Central Australia had similar frequency of cases across winter/summer seasons, whereas the Top End had significantly more during the dry season than the wet. Since 2009, the trend has reversed, indicating APSGN may not be reliably associated with seasonality in the NT.

Our study indicates that even though APSGN incidence has declined substantially around the world, there remains a high disease burden among the Aboriginal population in NT.^6,18,27,28^ Group A streptococcal infection and its sequelae diseases represent social disadvantage, poverty, remoteness, and overcrowding.^29^ Multiple approaches have been used in the past to tackle GAS infections and their sequelae with varying degrees of success. These include setting up community health-care programs that focus on prevention of skin diseases,^30,31^ establishing APSGN as a notifiable disease with subsequent public health action,^32^ and for acute rheumatic fever/rheumatic heart disease prevention providing secondary prophylaxis with long-acting penicillins.^33^ The reduction in worldwide prevalence of APSGN has coincided with improvement in living conditions and use of benzathine penicillin chemoprophylaxis for streptococcal pharyngitis in closed adult populations.^34–37^

Further planning is needed to build capacity in Aboriginal communities with community workers and clinicians to reduce the burden of GAS infection, to enhance data collection to look carefully at long-term outcomes of APSGN, and work to improve living conditions of the Aboriginal population. A multipronged approach to control GAS infection with improved hygiene, education, and living conditions of Aboriginal populations along with continued research into vaccine development is needed.
